# Development and validation of a continuous fall risk score in community-dwelling older people: an ecological approach

**DOI:** 10.1186/s12889-021-10813-w

**Published:** 2021-11-10

**Authors:** Jorge Bravo, Hugo Rosado, Pablo Tomas-Carus, Cristina Carrasco, Nuno Batalha, Hugo Folgado, Catarina Pereira

**Affiliations:** 1 Departamento de Desporto e Saúde, Escola de Saúde e Desenvolvimento Humano, Universidade de Évora, Largo dos Colegiais 2, Évora, Portugal; 2 Comprehensive Health Research Centre (CHRC), Universidade de Évora, Largo dos Colegiais 2, Évora, Portugal; 3 Department of Physiology, University of Extremadura, Badajoz, Spain

**Keywords:** Dynamic fall risk assessment, Fall prevention, Intrinsic-exposure risk, Older adults, Predictive accuracy

## Abstract

**Background:**

Fall risk assessment in older people is of major importance for providing adequate preventive measures. Current predictive models are mainly focused on intrinsic risk factors and do not adjust for contextual exposure. The validity and utility of continuous risk scores have already been demonstrated in clinical practice in several diseases. In this study, we aimed to develop and validate an intrinsic-exposure continuous fall risk score (cFRs) for community-dwelling older people through standardized residuals.

**Methods:**

Self-reported falls in the last year were recorded from 504 older persons (391 women: age 73.1 ± 6.5 years; 113 men: age 74.0 ± 6.1 years). Participants were categorized as occasional fallers (falls ≤1) or recurrent fallers (≥ 2 falls). The cFRs was derived for each participant by summing the standardized residuals (Z-scores) of the intrinsic fall risk factors and exposure factors. Receiver operating characteristic (ROC) analysis was used to determine the accuracy of the cFRs for identifying recurrent fallers.

**Results:**

The cFRs varied according to the number of reported falls; it was lowest in the group with no falls (− 1.66 ± 2.59), higher in the group with one fall (0.05 ± 3.13, p < 0.001), and highest in the group with recurrent fallers (2.82 ± 3.94, *p* < 0.001). The cFRs cutoff level yielding the maximal sensitivity and specificity for identifying recurrent fallers was 1.14, with an area under the ROC curve of 0.790 (95% confidence interval: 0.746–0.833; p < 0.001).

**Conclusions:**

The cFRs was shown to be a valid dynamic multifactorial fall risk assessment tool for epidemiological analyses and clinical practice. Moreover, the potential for the cFRs to become a widely used approach regarding fall prevention in community-dwelling older people was demonstrated, since it involves a holistic intrinsic-exposure approach to the phenomena. Further investigation is required to validate the cFRs with other samples since it is a sample-specific tool.

## Background

Beyond being the leading cause of injury-related death in older adults over age 65, falls induce significant restrictions on daily life activity, physiological condition deterioration, and impaired quality of life in this population [1]. Approximately one-third of this population who are living independently in the community falls at least once a year, and half of these individuals fall multiple times [2]. In addition to the direct impact on older people’s lives, falls and fractures also have a meaningful economic impact on health care systems worldwide [3]. Therefore, fall prevention in older people is a critical public health challenge, especially considering the collateral impact that a fall episode in a care recipient might have on the informal caregiver’s life [4].

Effective fall prevention strategies benefit from a more comprehensive understanding of the causal falling mechanisms; thus, a comprehensive fall risk assessment is crucial to design effective fall prevention programs. Moreover, a screening protocol should be short, easy to administer, and multifactorial, allowing the identification of the risk factors that can be targeted by an intervention [5, 6]. Medical associations and national health authorities are unanimous in recommending the use of fall risk assessment tools [7]. Hence, several reviews reporting fall risk assessment instruments and their accuracy are available in the literature [8, 9]. Fall risk assessment instruments commonly include risk factors such as previous fall occurrence, impaired balance and gait, and medical conditions such as chronic diseases, physical impairments, and medication use [10]. Nevertheless, current assessment instruments are considered inadequate or limited in terms of predicting falls in community-dwelling older people; thus, researchers recommend that fall risk assessment tools should not be used in isolation to identify older people at high risk of falls [9, 11].

Fall risk models based on the dynamic interplay between intrinsic risk factors that vary over time and exposure (activity in a context that creates the opportunity for fall occurrence) were recently proposed to increase assessment accuracy [6]. The rationale for this approach contradicts the mistaken assumption that risk factors are stable for each person and do not change over time. However, fall risk factors evolve and regress during the life cycle and are interconnected in a dynamic interplay. A change in any of the living conditions (e.g., mobility restriction, altered medication, or a fall event) may affect other risk factors, so models for assessing the risk of falls should be equally dynamic [6]. As an example, a fall risk assessment tool based on a medical and physiological theoretical framework proved to be valid, reliable, and feasible to predict multiple falls; nonetheless, the risk cutoffs derived from dichotomous classifications [12] limited its use across all ages and different populations.

Dynamic approaches to risk factors based on continuous scores have been widely used in cardiometabolic risk factors [13, 14]. Continuous scoring is considered a method that better adjusts for progressively increased risk with an increasing number of risk factors and is a valuable tool for epidemiological analyses, making it useful for researchers, clinicians, and policymakers [15]. A dynamic fall risk model derived from a continuous score based on intrinsic and exposure risk factors, which is a sample-specific multifactorial dynamic approach, would provide a better understanding of the complex interaction among fall risk factors. This method might help researchers and practitioners develop more individualized prevention strategies and allow individualized control of older people’s risk progression over time. Furthermore, it seems to be an easy-to-use, reproducible, and quick method to assess fall risk.

To the best of our knowledge, no study has developed a dynamic fall risk model based on a continuous intrinsic-exposure risk score to assess the risk of falls in community-dwelling older people. This study primarily aimed to develop and examine the construct validity of a dynamic fall risk model based on an intrinsic-exposure multifactorial sex-specific continuous fall risk score (cFRs) for Portuguese community-dwelling older people. The secondary purpose was to identify the cFRs cutoff discriminating those at low and high risk for multiple falls.

## Methods

### Study design and participants

This study follows an observational cross-sectional design. Participants were recruited from senior universities, parishes, city halls, and senior associations through leaflet and poster distributions. The inclusion criteria were as follows: older people aged ≥65 years from the Alentejo region (Portugal) living independently in the community, without cognitive impairment (scoring > 24 points in the Folstein Mini-Mental State Examination) [16]. Respondents were excluded if they experienced a recent acute health event (e.g., myocardial infarction or fractures leading to immobilization). The authors calculated the minimum representative sample size of this population using the national census [17] data concerning older people ≥65 years old (n = 182,988), and the epidemiologic statistical OpenEpi software [18]. The calculated minimum sample size was 384 older people, considering a 95% significance level (Z_a_ = 1.96), a 5% sampling error, the population dimension, and a 50% hypothesized frequency of the outcome factor in the population (p). Five hundred and eight older persons volunteered for the study. Due to the presence of cognitive impairment, four respondents were excluded from our analysis, resulting in a total of 504 participants enrolled in the study (391 women: 73.1 ± 6.5 years; 113 men: 74.0 ± 6.1 years). All participants reviewed and signed an informed consent form. The study was approved by the University of Évora Ethics Committee for research in the areas of human health and well-being (reference number 16–012).

### Data collection

The data collection took place between April 2017 and January 2018 at the Superior Nursing School Laboratory in Évora, Portugal. Face-to-face interviews were conducted by four specialized raters who were blind to the study’s objectives to collect sociodemographic data (sex, age) and self-reported clinical history, including the number of chronic diseases, physical impairments and previous fall occurrences. To maintain anonymity, a code was assigned to each participant during data collection.

### Measures

#### Falls

Falls were defined as “an unexpected event in which the participants come to rest on the ground, floor, or lower-level” [19]. Only falls resulting from ordinary daily life activities were considered, and falls resulting from risky and dangerous circumstances or traffic accidents were excluded. Fall episodes within the previous 12 months were assessed during the interview. The participants were categorized into two groups: occasional fallers and recurrent fallers. Occasional fallers were defined as those who fell once or who had not fallen in the 12 months preceding the evaluation, and recurrent fallers were defined as those who fell twice or more in the same period. Fall-related consequences and the detailed description of each fall were captured to ensure an accurate number of previous falls.

#### Body composition

Body composition was assessed through standing height and weight measured using a stadiometer (Seca 770, Hamburg, Germany) and an electronic scale (Seca Bella 840, Hamburg, Germany), respectively, to compute body mass index (m/kg^2^). Waist circumference was measured using anthropometric tape following World Health Organization procedures (midway between the lower rib margin and the iliac crest) [20].

#### Chronic diseases

Chronic diseases were derived from the 24 chronic comorbidities listed by all participants, including metabolic disease, diabetes mellitus, cardiovascular disease, respiratory disease, stroke, neurological disorders, peripheral vascular disease, renal insufficiency, and arthritis. The “chronic diseases” variable corresponds to the number of chronic comorbidities present in each participant.

#### Physical impairments

The physical impairments variable was calculated by summing the reported impairments, including frequent dizziness, involuntary loss of urine, poor vision, foot problems, hearing problems and occasional loss of balance [21]. Chronic diseases and physical impairment information was double-checked with the records from the medical clinical report and current medication type and dosage.

#### Cognitive impairments

Cognitive impairments resulted from the participants’ score on the Portuguese version of the Mini-Mental State Examination [16, 22], a 30-item test divided into the following components: orientation, registration, attention, delayed recall, language and praxis. Mini-Mental State Examination scores range from 0 (worst) to 30 (best) points.

#### Physical function

Physical function was assessed by the 12-item responses to the Composite Physical Function (CPF) Scale [23], based on the participants’ perception of whether they “could perform the activity” (score 2), “could perform it with difficulty or with help” (score 1) or “could not perform the activity at all” (score 0). In this study, the physical function variable represents the total CPF score, ranging between 0 (worst) and 24 (best) points. Multidimensional balance was evaluated by the sum of points obtained in each of the ten tests of the Fullerton Advanced Balance Scale [24], scoring each test between 0 (worst) and 4 (best), into a total grade ranging between 0 and 40 points. Fear of falling was assessed by the modified Falls Efficacy Scale - International (FES-I) used by Yardley et al. [25] and originally developed by Tinetti et al. [26]. Participants were asked how concerned they felt, on a scale from 1 (not concerned) to 4 (very concerned), about falling while performing each of the sixteen everyday activities listed in the FES-I, generating a total score ranging from 16 to 64 points [25].

Affordance perception, an indicator of individual perception of stepping-forward boundaries, was assessed through the stepping-forward affordance perception test; the test protocol has been described in detail elsewhere [27]. The estimated stepping-forward (cm) and real stepping-forward (cm) distances were measured, and posterior computation was performed to produce the absolute error (|real stepping-forward – estimated stepping-forward|), which was the main representative outcome of affordance perception accuracy [27]. An underestimated absolute error works as a protective mechanism for fall occurrence.

Changes in walking performance were measured through the Gait Section of the Tinetti’s Performance-oriented Assessment of Mobility [28, 29]. This instrument assesses gait based on eight items. A complete description of gait assessment items and on how scores are assigned can be found elsewhere [28, 30]. The “gait score” ranged from 0 (worst) to 12 (best).

#### Physical activity

Habitual physical activity was assessed by the short version of the International Physical Activity Questionnaire [31]. Participants are asked about the time spent walking and participating in moderate and vigorous activities to calculate their total metabolic expenditure (metabolic equivalent of tasks: MET-min/week). The calculation considered the time spent (min/week), the frequency (days/week) and the intensities of each physical activity type: walking (3.3 MET), moderate activity (4.0 MET) and vigorous activity (8.0 MET).

#### Environmental hazards

Both the interior and exterior of the participants’ dwellings were checked for environmental hazards, including animals, stairs, raised carpets, and habitual footwear. The presence of each listed environmental hazard was checked for each participant, and the total number of hazards was counted (minimum: 0, maximum: 34) [32].

### Derivation of the continuous fall risk score

The cFRs considers intrinsic and exposure fall risk factors. Intrinsic risk factors included chronic diseases, physical impairments, cognitive impairments, physical function, multidimensional balance, fear of falling, affordance perception, and gait. Exposure took into consideration habitual physical activity, particularly the total metabolic expenditure throughout a typical week, and the environmental hazards identified in each participant’s traditional environment. These two exposure measures are concerned with the opportunity for fall occurrence. Both measures integration was recently proposed in a dynamic fall risk models’ conceptualization to characterize the exposure, considering the context (activity type) of the fall event and the environment (environmental hazards) in which the activity was performed [6]. Therefore, supported by their recognized relationship with the risk of falling [10, 33–42], the above controllable intrinsic and exposure fall risk factors were included in the cFRs.

The cFRs was derived from the sum of the standardized residuals (Z-scores) of each controllable intrinsic and exposure fall risk factor. Computation of Z-scores for each key component was performed by regressing them onto age and sex to account for any age- and sex-related differences. Because cognitive impairments, physical function, multidimensional balance, affordance perception, gait, and physical activity Z-scores are inversely related to fall risk, they were multiplied by − 1 before Z-score summation. A higher cFRs indicates a higher risk of falling.

### Statistical analysis

The data contained some missing values in the variables used to compose the cFRs with no obvious patterns across variables and participants, following a missing at random mechanism. Approximately 24.3% of the participants would be lost after listwise deletion of the data if we decided to keep only the participants with no missing values on any items. Therefore, missing values were replaced using the fully conditional specification imputation method [43]. Eight imputations were created from the original dataset to generate plausible values drawn from a predicted distribution on the basis of other observed variables using a multiple imputation software package (SPSS version 24.0, IMB Statistics). Multivariate imputation accounting for missingness in the cFRs variables was as follows: cognitive impairments (1.3%), physical function (2.8%), multidimensional balance (1.1%), fear of falling (1.7%), affordance perception (8.5%), gait (4.3%) and physical activity (4.6%).

Other statistical analyses were performed using the *R* and *Jamovi* software packages [44, 45]. The percentage of occasional and recurrent fallers was calculated for the total sample and stratified by sex. Descriptive statistics were performed for body composition and for the cFRs components considering the total sample and stratified by fall status (occasional and recurrent fallers). Comparisons between participants according to fall status were performed in numeric variables by the use of independent sample t-tests, whereas proportion comparisons were performed by Pearson χ^2^ tests (p < 0.05). Effect sizes were quantified for the pairwise comparisons between fall status for continuous variables to indicate the practical meaningfulness of the mean value differences, shown as Cohen’s d with 90% confidence intervals. Thresholds for effect sizes based on Batterham and Hopkins guidelines [46] were trivial (< 0.2), small (0.2 to < 0.6), moderate (0.6 to < 1.2), large (1.2 to < 2.0), and very large (> 2.0).

One-way analysis of variance with Tukey’s post hoc test was used to analyze how the cFRs varied according to the reported number of falls over the previous 12 months (0 falls, one fall, and two or more falls). Receiver operating characteristic (ROC) analysis was used to determine the sample-specific cutoff point that minimized the total number of misclassification errors and to investigate the accuracy of the cFRs and each key component in discriminating recurrent fallers. A dichotomous classification as recurrent faller (yes = 1; no = 0) was used in the ROC analysis. Sensitivity was defined as the percentage of recurrent fallers who were correctly identified by the estimated cFRs cutoff point, and specificity was defined as the percentage of occasional fallers who were correctly identified by the estimated cFRs cutoff point. The optimal cutoff score for each key component and the cFRs were determined using Youden’s method [47]. The positive predictive value was defined as the percentage of subjects who were recurrent fallers (those with cFRs scores equal to or above the cutoff point), and the negative predictive value was the percentage of subjects who were occasional fallers (those with cFRs scores below the cutoff point). Positive and negative predictive values were calculated for each cFRs key component and the total cFRs, as well as their associated 95% confidence intervals, using bootstrapping. Sensitivity and specificity 95% confidence intervals were calculated using the free online VassarStats Clinical Calculator 1 (http://vassarstats.net/clin1.html).

The area under the curve (AUC) was used to measure the accuracy of the cFRs and each key component to discriminate occasional and recurrent fallers [48]. In this analysis, the AUC can be considered equivalent to the probability that a randomly drawn recurrent faller has a higher cFRs than a randomly drawn occasional faller. The AUC was interpreted according to the following guidelines: noninformative/test equal to chance (AUC = 0.5), less accurate (0.5 < AUC ≤ 0.7), moderately accurate (0.7 < AUC ≤ 0.9), highly accurate (0.9 < AUC ≤ 1.0), and perfect discriminatory tests (AUC = 1.0) [49].

## Results

Out of the entire sample (n = 504), 148 participants (29.4%) had fallen twice or more over the previous 12 months and were defined as recurrent fallers; among them, 118 (23.4%) were females and 30 (6.0%) were males. Most participants (70.6%) had fallen once or less over the same period and were considered occasional fallers. The overall sample characteristics were stratified by occasional and recurrent fallers and are displayed in Table 1. There were no significant differences in the mean age or body composition between occasional and recurrent fallers. The recurrent fallers reported significantly more chronic diseases (3.3 vs. 2.2 total, p < 0.001, Cohen’s *d* = 0.60) and physical impairments (4.0 vs. 2.2 total, p < 0.001, Cohen’s *d* = 1.03) than occasional fallers. Compared with recurrent fallers, occasional fallers had a higher performance on balance (30.3 vs. 26.5 score, p < 0.001, Cohen’s *d* = − 0.54) and gait (11.6 vs. 11.1 score, *p* < 0.001, Cohen’s *d* = − 0.37) tests and reported a higher functional capacity (21.2 vs. 19.1 score, p < 0.001, Cohen’s *d* = − 0.52). Occasional fallers also revealed fewer cognitive impairments (27.0 vs. 22.2 score, p < 0.001, Cohen’s *d* = − 1.36), reported less fear of falling (20.1 vs. 25.9 score, *p* < 0.001, Cohen’s *d* = 0.93) and identified more environmental hazards in their environment (11.2 vs. 9.5 score, *p* < 0.001, Cohen’s *d* = − 0.26). The cFRs was significantly higher in recurrent fallers than in occasional fallers (− 1.24 vs. 2.99 scores, p < 0.001, Cohen’s *d* = 1.21). The mean for the total sample was 0.00 ± 4.0.

**Table 1:** Characteristics of the total sample and by the fall status (occasional and recurrent fallers)

Variables^**a**^	Entire sample	Occasional fallers	Recurrent fallers	***P***-value	Cohens’ d (90% CI)
	***n*** = 504	***n*** = 356 (70.6%)	***n*** = 148 (29.4%)		
Age (years)	73.3 ± 6.4	73.1 ± 6.4	73.8 ± 6.5	0.236	0.11 (− 0.10;0.27)
Female (n/%)	391 (77.6%)	273 (54.2%)	118 (23.4%)	< .001	−0.27 (− 0.33;-0.20)
Male (n/%)	113 (22.4%)	83 (16.5%)	30 (6.0%)	< .001	−0.30 (− 0.34;-0.26)
Body composition					
Weight (Kg)	69.9 ± 11.7	70.0 ± 11.9	69.5 ± 11.3	0.636	−0.05 (− 0.20;0.12)
Height (cm)	155.7 ± 8.4	155.8 ± 8.6	155.4 ± 7.7	0.596	−0.05 (− 0.21;0.11)
Waist circumference (cm)	96.7 ± 10.9	97.0 ± 10.9	96.1 ± 11.0	0.406	−0.08 (− 0.24;0.08)
Body mass index (Kg/m^2^)	28.8 ± 4.1	28.8 ± 4.1	28.7 ± 4.0	0.869	−0.03 (− 0.19;0.14)
Chronic diseases (total)	2.5 ± 1.8	2.2 ± 1.6	3.3 ± 2.2	< .001	0.60 (0.45;0.78)
Physical impairments (total)	2.9 ± 1.7	2.4 ± 1.5	4.0 ± 1.7	< .001	1.03 (0.86;1.19)
Cognitive impairments (score)	26.5 ± 3.6	27.0 ± 3.2	22.2 ± 4.2	< .001	−1.36 (− 1.54;-1.19)
Physical function (score)	20.6 ± 4.1	21.2 ± 3.5	19.1 ± 5.1	< .001	−0.52 (− 0.68;-0.36)
Multidimensional balance (score)	29.2 ± 7.3	30.3 ± 6.1	26.5 ± 9.0	< .001	−0.54 (− 0.70;-0.37)
Fear of falling (score)	21.8 ± 6.7	20.1 ± 4.9	25.9 ± 8.6	< .001	0.93 (0.77;1.10)
Affordances perception (cm)	8.3 ± 7.2	8.5 ± 7.3	7.9 ± 7.0	0.429	−0.08 (− 0.24;0.08)
Gait (score)	11.4 ± 1.4	11.6 ± 1.1	11.1 ± 1.8	< .001	−0.37 (− 0.53;-0.21)
Physical activity (Mets/week)	1996.5 ± 1928.1	2069.5 ± 1933.7	1753.6 ± 1712.9	0.085	−0.17 (− 0.33;-0.01)
Environmental hazards (total)	10.7 ± 6.6	11.2 ± 6.5	9.5 ± 6.8	0.010	−0.26 (− 0.42;-0.10)
cFRs (standardized score)	0.00 ± 4.0	−1.24 ± 3.2	2.99 ± 4.1	< .001	1.21 (1.04;1.39)

^a^Data are mean ± standard deviation or n (%). *METs* metabolic equivalent of tasks, *cFRs* continuous fall risk score, *CI* confidence intervals

**Fig. 1:**
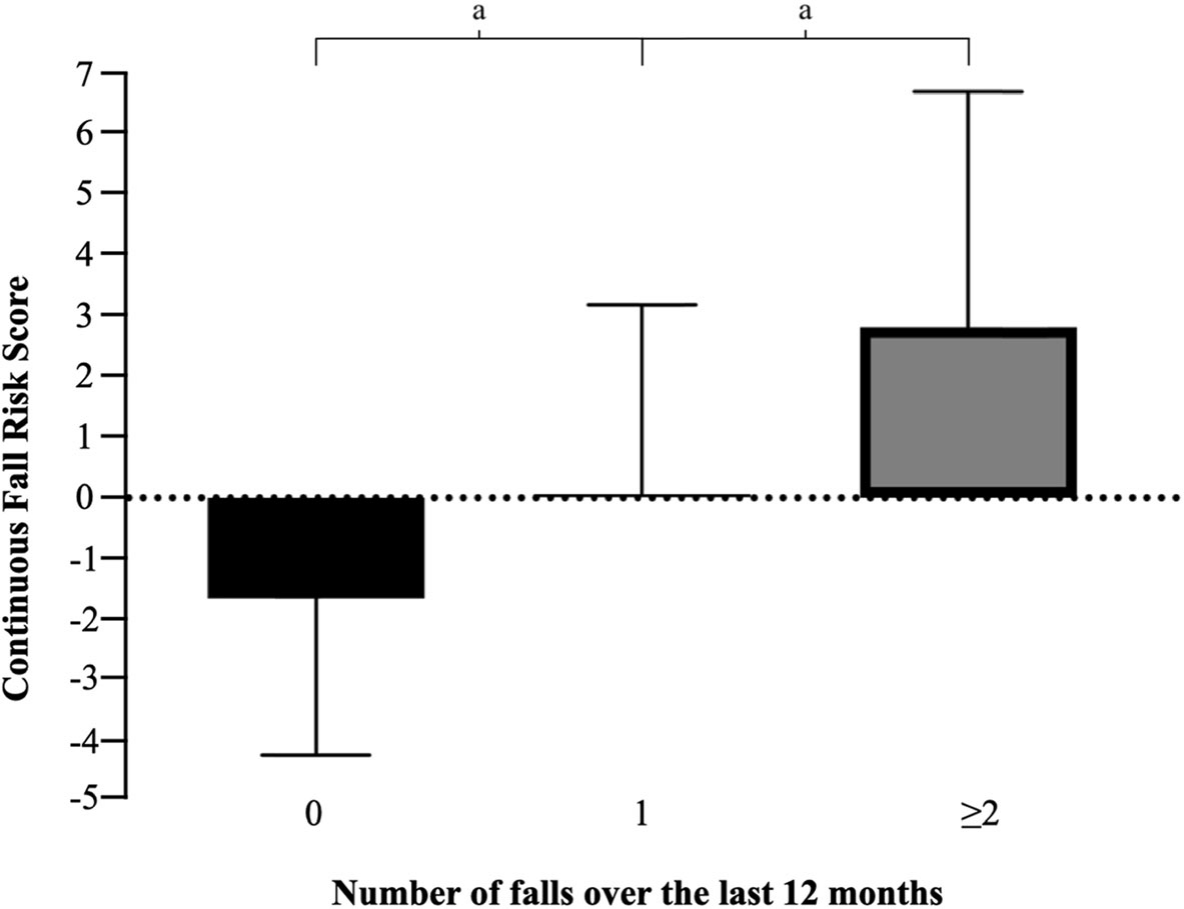
Continuous Fall Risk Score by the number of falls over the last 12 months (0 falls, 1 fall, and two or more falls). ^a^*p* < 0.001

Figure 1 shows how the cFRs varies according to the reported number of falls over the previous 12 months (0 falls, one fall, and two or more falls). There were significant differences between all fall groups (*p* < 0.001); the cFRs was lowest in the group with no falls (− 1.66 ± 2.59), higher in the group with one fall (0.05 ± 3.13), and highest (2.82 ± 3.94) in those who fell twice or more over the last 12 months (recurrent fallers).

**Fig. 2:**
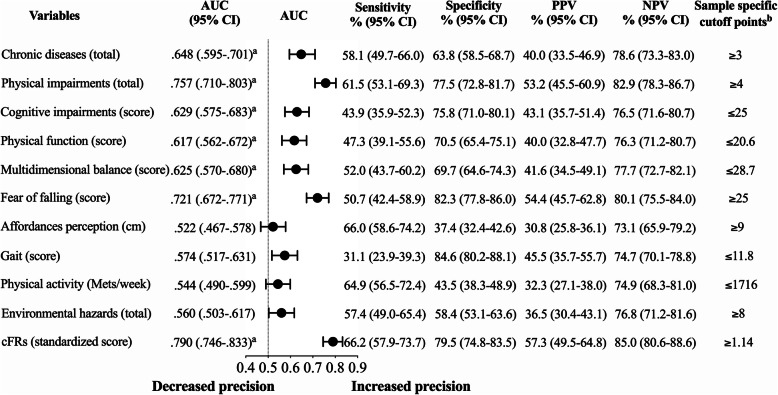
Continuous fall risk score and key components sample specific cutoff points and respective accuracy in discriminating recurrent fallers. ^a^*p* < 0.001. ^b^The point of intersection between lines of specificity and sensitivity identified the highest numbers of participants who reported 2 or more falls

Figure 2 presents the findings of the ROC curve analyses providing sample-specific cutoffs for the cFRs and its key components, yielding the maximal sensitivity and specificity (Youden’s index) to identify recurrent fallers accurately. The cutoff yielding the highest Youden’s index for identifying recurrent fallers was a cFRs of 1.14, with an AUC = 0.790 (0.746–0.833, 95% confidence intervals) (p < 0.001), indicating that the cFRs was a moderately accurate method (0.7 < AUC ≤ 0.9) to identify recurrent fallers. Key component sample-specific cutoffs and their corresponding sensitivity and specificity are also provided in Fig. 2 for interpretation by the reader.

**Fig. 3:**
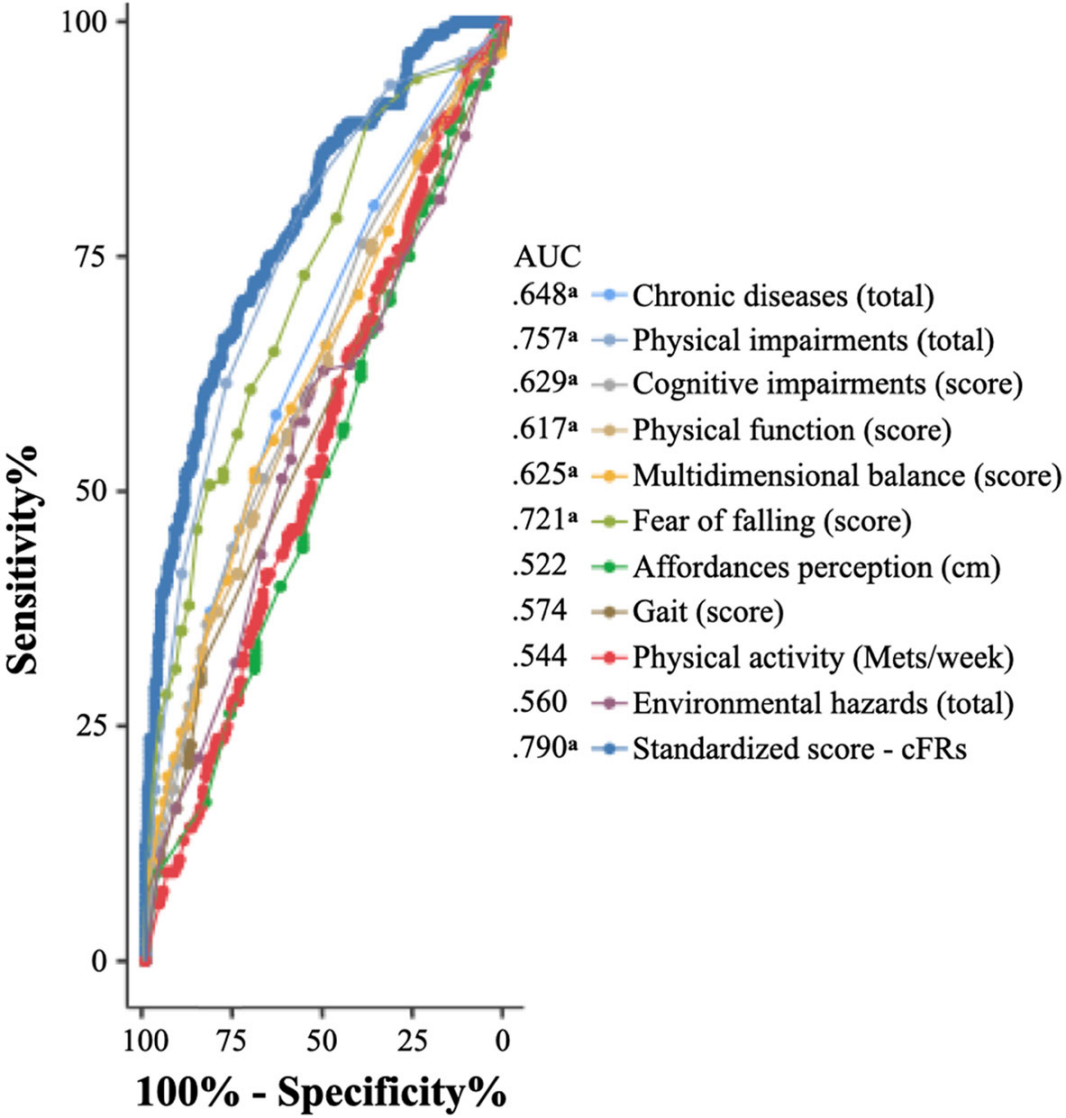
Receiver operating characteristic curve for each key component and for the continuous fall risk score as predictors of being a recurrent faller. ^a^*p* < 0.001

Figure 3 represents the overall ROC curves for the cFRs and its key components. According to the ROC analyses, the cFRs performed reasonably better in identifying recurrent fallers. The cFRs revealed the highest AUC = 0.790 (0.746–0.833, 95% confidence intervals) (p < 0.001) when compared to each controllable intrinsic and exposure fall risk factor, which varied between an AUC = 0.522 (0.467–0.578, 95% confidence intervals) for affordance perception and an AUC = 0.757 (0.710–0.803, 95% confidence intervals) (*p* < 0.001) for physical impairments.

## Discussion

In this study, we developed and validated the cFRs, a dynamic fall risk model based on an intrinsic-exposure multifactorial sex-specific continuous score, to assess the risk of falls in Portuguese community-dwelling older people. To our knowledge, this is the first investigation to use a sex-specific and intrinsic-exposure multifactorial dynamic approach to determine fall risk in a representative sample of older people. We have further investigated the cFRs accuracy for identifying recurrent fallers, and our main finding is that the cFRs has construct and predictive validity to identify recurrent fallers in this population. The cFRs revealed an AUC of 0.790, which indicates that 79% of the older persons were classified correctly by using this score. Moreover, our results showed that the cFRs varied according to previous fall episodes; the cFRs mean value increased with an increasing number of previous falls. The optimal cFRs cutoff point to identify recurrent fallers was > 1.14, with sensitivity and specificity values of > 66.2% and > 79.5%, respectively.

The use of continuous scores to assess cardiovascular and metabolic risk is quite common, mainly because these pathologies result from a set of multifactorial risk factors, including obesity, lipid profile, glucose levels or insulin resistance, blood pressure and other lifestyle components such as smoking habits and physical inactivity [14, 15]. Several statistical approaches, including principal component analysis, standardized residuals of Z-scores, and percentile rankings, have been applied due to the diversity of the variables included in the risk score calculation [13, 50, 51]. Similarly, prior fall risk assessments are not generalizable, mostly due to the enormous diversity of risk factors contributing to the occurrence of falls, which makes it impossible to compare fall risk prevalence between studies. Therefore, modeling the relationship between the various risk factors by using traditional statistical methods may overcome these limitations. To our knowledge, this is the first study that has developed a continuous score for fall risk assessment in older people. Despite the utility of the cFRs in epidemiological research, we recognize that the accuracy levels of a continuous score to assess the risk of falls can hardly come close to the accuracy levels of continuous scores developed to assess cardiovascular and metabolic risk [51]. Cardiovascular and metabolic risk factors are mainly supported by precise physiological indicators such as the lipid profile, blood pressure or glucose levels, generating more precise data to include in the statistical models. Thus, continuous cardiovascular and metabolic risk algorithms regularly reach moderate predictive accuracy (between 0.7 and 0.9), while fall risk predictive models rarely reach a moderate predictive capacity [6].

Watchful to the need to develop multifactorial fall risk predictive models, some authors reported a low predictive capacity of predictive models with an AUC below 0.650 for occasional fallers [52] and 0.720 for recurrent fallers [12]. The results of the cFRs developed in this study revealed a moderate predictive capacity to identify recurrent fallers, with an AUC of 0.790. In their research, Tiedemann et al. [12] first developed and validated a fall risk assessment tool for use in primary care with community-living older people to discriminate between multiple fallers and non–multiple fallers with an accuracy of 72%. Despite being the most similar approach to the one used in our study, the model proposed by Tiedemann et al. [12] used intrinsic fall risk factors exclusively. In our study, individual exposures (activity and environmental context) were also assessed. The creation of predictive models for fall occurrence in older people that include exposure to hazardous situations was recently proposed by Klenk et al. [6]. The authors support the assessment of environmental hazards and activity levels, since the individual intrinsic risk for falls may be present but it is the personal exposure that creates the opportunity for the fall occurrence [6]. Nevertheless, in the method proposed by Tiedemann et al. [12], the development of the predictive model was performed based on the dichotomization of the study variables while looking for the assessment measure cutoff values and limiting its use to specific ages and populations. In contrast, in our study, a continuous risk score was generated, which we believe to be a dynamic and ecological method [6] to study falling phenomena; this approach is usable across all ages and with different populations.

Some researchers consider that a dynamic framework for a fall risk model can prompt greater awareness of contextual and environmental aspects in for clinicians, older people, and families, providing them with a comprehensive picture of the person who falls and the mechanisms behind the fall [6]. In their study procedure validation, Palumbo et al. [5] found that the most frequently selected variables included in models explaining falls were intrinsic risk factors, namely, the history of falls, self-perceived health status, depression, number of medications, and the use of drugs acting on the central nervous system. Reinforcing the multifactorial etiology of falls, Palumbo et al. [5] performed Lasso model accuracy-parsimony analysis and revealed that predictive accuracy improved with 20 or more variables. Compared to our cFRs (AUC = 0.790), Palumbo et al.’s [5] predictive model accuracy was lower (AUC = 0.708), despite being a moderately accurate method for fall prediction based on prospective fall occurrence. Although Tiedemann et al. [12] and Palumbo et al. [5] validated their models against prospective fall occurrence, in our study, we chose to validate the cFRs against retrospective fall occurrence. Aware of the recall bias risk when assessing retrospective falls, our approach is supported by some investigations suggesting that a single visit interview can be as accurate as a costly 12-month prospective approach to evaluate the incidence of falls [53]. We further assume that the regular checks of fall incidence over time performed in prospective approaches may influence older people’s behaviors, working as a constant reminder and resulting in a preventive intervention rather than an evaluation process.

Another advantage of our sex-specific and intrinsic-exposure multifactorial dynamic approach to determine fall risk is that this approach made it possible to find a cutoff value (cFRs = 1.14) differentiating people who have a low or high risk of falls, which, in turn, makes it possible to identify the priority population that may benefit from a fall prevention program. This is of extreme importance because many older people, caregivers, or even health professionals are not aware of the individual risk of falling [54, 55].

Although we recognize the advantages of an ecological approach to the risk of fall assessment supported by intrinsic risk factors and individual exposure to risk, we highlight the moderate predictive capacity revealed by some instruments used in the present study for the cFRs calculation. For example, physical activity assessed by the IPAQ only showed a low capacity to predict the risk of falling in older people, since the computed AUC was 0.544. However, the physical impairments and fear of falling assessment instruments proved to be moderately accurate tools to predict the risk of falling in older people, with AUCs of 0.757 and 0.721, respectively, which were still lower than that of the cFRs (AUC = 0.790). In line with our findings, other studies have reported a lower predictive accuracy of isolated risk factors (AUC < 0.700) [56] compared to multifactorial models (AUC > 0.700) [12].

Our results have practical implications concerning fall prevention. Any healthcare professional or even an informed caregiver can easily perform the fall risk evaluation. Calculating the cFRs enables the caregiver to compare the risk score against the scores of other older people or against own previous scores, which is particularly useful for intervention program control. The cFRs should be seen as a dynamic fall risk model that gives researchers, older people, and caregivers a structured and continuous perspective of individual fall risk scores that may lead to new advances in fall prevention. An older person’s cFRs can be followed over time as a whole system, independent of improvements or worsening in specific risk factors that may affect more or less the entire system. Considering this ecological approach based on intrinsic-exposure dynamic fall risk models, we may expect that, in the future, the predictive accuracy will increase with the inclusion of data from body-worn sensors and other technological wearable sensor units that may continuously measure intrinsic and exposure parameters [57].

Some limitations must be acknowledged in the interpretation of the results of this study. First, despite the consensus that fall risk factors in older people are multifactorial and change over time, there is no consensus on the risk factors determining fall occurrence or on their relative importance. Second, the cFRs is sample-specific, hence the need for future research to validate its usefulness in older people across different populations. Even so, this standardized method of calculating a continuous score may be beneficial for comparing epidemiological studies. Third, the cFRs is based upon the assumption that each selected variable is equally important in defining fall risk determination. However, some variables may have a greater weight in explaining fall risk. Furthermore, an older person being described as “at risk” does not necessarily mean that the person is at high risk for all fall risk factors. We challenge researchers and practitioners to investigate the application of other statistical techniques that select the principal components for assessing the risk of falling based on the underlying relationship between fall risk factors, such as principal component analysis.

## Conclusions

The cFRs was shown to be a valid dynamic multifactorial fall risk assessment tool to identify older people who experience recurrent falls. This holistic intrinsic-exposure approach to fall risk assessment represents a valid alternative for epidemiological analyses and clinical practice, showing its potential to become a widely used approach regarding fall prevention in community-dwelling older people. In addition, this method allowed us to establish the cutoff value identifying Portuguese older persons at high risk of recurrent falls: cFRs > 1.14. Further investigation is required to validate the cFRs with other samples since it is a sample-specific tool.

## Data Availability

The datasets used and/or analyzed for the current study are available from the corresponding author upon reasonable request.

## Abbreviations

cFRs: Continuous Fall Risk Score
CPF: Composite Physical Function
FES: Falls Efficacy Scale
MET: Metabolic equivalent of task
ROC: Receiver operating characteristics
AUC: Area under the ROC curve
SPSS: Statistical Package for the Social Sciences
IBM: International Business Machines Corporation
CI: Confidence intervals
PPV: Positive Predictive Value
NPV: Negative Predictive Value
